# Knowledge and Perception on Long Acting and Permanent Contraceptive Methods in Adigrat Town, Tigray, Northern Ethiopia: A Qualitative Study

**DOI:** 10.1155/2014/878639

**Published:** 2014-07-21

**Authors:** Alem Gebremariam, Adamu Addissie

**Affiliations:** ^1^Department of Public Health, College of Medicine and Health Sciences, Adigrat University, P.O. Box 50, Adigrat, 1029 Tigray, Ethiopia; ^2^School of Public Health, College of Health Sciences, Addis Ababa University, Addis Ababa, Ethiopia

## Abstract

*Background*. Long acting and permanent contraceptive methods have the potential to reduce unintended pregnancies but the contraceptive choice and utilization in Ethiopia are highly dominated by short term contraceptives. *Objective*. To assess the knowledge and perception on long acting and permanent contraceptives of married women and men in Northern Ethiopia. *Method*. A qualitative method was conducted in Adigrat on January, 2012. Four focus group discussions with married women and men and six in-depth interviews with family planning providers were conducted. Content analysis was used to synthesize the data. *Result*. Participants' knowledge on long acting and permanent contraceptives is limited to recognizing the name of the methods. Most of the participants are not able to identify permanent methods as a method of contraception. They lack basic information on how these methods work and how they can use it. Women had fears and rumors about each of these methods. They prefer methods which do not require any procedure. Family planning providers stated as they have weakness on counseling of all contraceptive choices. *Conclusion*. There are personal barriers and knowledge gaps on these contraceptive methods. Improving the counseling service program can help women to increase knowledge and avoid misconceptions of each contraceptive choice.

## 1. Background

Family planning (FP) is a process that usually involves a discussion between a woman, a man, and a trained FP service provider focusing on family health and the desires of the couple to either limit or space their family [1]. There are different methods used for FP. Contraceptive methods used for FP can be grouped into two categories programmatically. These are long-acting and permanent methods (intrauterine devices, implants, and sterilization) and short-term methods (pills, condoms, spermicides, injectables, other modern methods, and all traditional methods). Long-acting and permanent methods are usually used to limit childbearing, whereas short-term methods are better suited for women who want to delay but not forfeit having a child [2].

The total fertility rate of Ethiopia is 4.8 children per women. A great majority of the health facilities in Ethiopia offer oral pills (98.8%) and injectables (98.0%) followed by male condom (95.2%), implants (75.0%), IUDs (53.6%), female sterilization (22.6%), male sterilization (16.7%), and female condom (4.0%) [3]. The modern contraceptive prevalence rate among currently married women is 29%. The contraceptive method mix is highly dominated by short-term contraceptive methods. Thirty-seven percent of women want no more children but only 3.4% of married women reported using implants, 0.3% IUD, and less than 1% as sterilized [4]. The overall prevalence of long acting and permanent contraceptive methods (LAPMs) was 12.3%. There were no users for female or male sterilization. Majority of the participants (93.3%) stated that using another method of contraception is their main reason for not using LAPMs. A significant amount of the participants had low knowledge on permanent contraceptive methods, particularly vasectomy. More than half (53.6%) of married women had negative attitude towards practicing of LAPMs [5].

Women had different perceptions on modern contraception. Some women had expressed their fear on contraceptive methods. They said it might cause excessive bleeding, infertility, or cancer [6, 7]. Misinformation about modern contraceptive methods is still present. There was a widespread belief that the intrauterine device (IUD) makes the user more prone to sexually transmitted infections (STIs) and infections of the pelvis [6]. Shifting or expulsion of IUD or implants was cited by a number of participants. Some women perceive that implants could get lost in the body via the blood stream. Similarly, they perceive that IUD could shift during sexual intercourse with serious implications for birth outcomes [7].

Information on women's and men's knowledge on contraceptive methods provides a measure of awareness of contraception in the population and indicates the success of information, education, and communication programmes. In addition, knowledge of at least one modern contraceptive method and a positive attitude towards that contraceptive method are prerequisites for the use of that contraceptive [4]. The objective of this study was to determine knowledge and perception regarding LAPMs among married women and men. The findings of this assessment will help in designing relevant and culturally appropriate interventions that will aid the efforts at promoting and disseminating information on LAPMs to the eligible individuals and couples.

## 2. Methods

This study was a qualitative study conducted in Adigrat town, Northern Ethiopia. The study took place on January 2012. Four focus-group discussion (FGD) sessions with purposively selected three groups of married women and one group of married men were conducted. Separate male and female FGDs were seen as the preferred protocol in order to discuss FP and reproductive health, which are considered private and sensitive topics. A purposive sampling approach was employed to recruit 32 participants of the target group consisted of seven up to twelve discussants. Both men and women had been identified and the participants were recruited through community leader contacts. The most commonly used form of nonprobabilistic sampling is purposive sampling and their size relies on the concept of “saturation,” or the point at which no new information or themes are observed in the data or cutoff between adding emerging findings and not adding or when the researcher is no longer hearing or seeing new information [8]. The inclusion criteria were married men and women, with ages between 15 and 49 years for women and 18 and 65 years for men.

FGD guide was used for eliciting information including the following topics such as social, demographic issues, knowledge and practice of modern contraceptives, method preference, advantages and disadvantages of LAPMs, barriers and perceptions about FP, community attitude and perception concerning the use of LAPMs, and suggestions.

A total of six in-depth interviews were conducted with purposively selected family planning (FP) service providers to see the perceptions and choice of clients towards LAPMs. The inclusion criterion was health professionals in the health facilities who offer any of the LAPMs and were working during the data collection period. Interview guide was used to extract information about training status of the provider; capacity of counseling and providing long acting and permanent contraceptive methods; women's knowledge, perception, and preference of modern contraceptives in their clinic; what could improve the provision of family planning services in particular LAPMs; and suggestions.

After selecting the participants, appropriate time and comfortable place of meeting was selected. Discussions and interviews were conducted in the local languages (Tigrigigna). All study participants were encouraged to openly discuss their opinions. Discussion continued until information saturation is reached. The FGDs and in-depth interviews were taped and note was taken. Audio data were transcribed verbatim into Microsoft Word files and translated from the local language to English. The principal investigator and note taker reviewed key terms in local language and their respective translations to ensure a degree of standardization. A small, selected sample of interviews was translated from Tigrigna into English by an independent translator to assess the accuracy of the translation. Final transcripts were compared against note takers' notes to ensure quality. Before the analysis, the text was read through several times to obtain a sense of the whole and familiarize with the data. The data was first saved in text format and imported in to open code software version 3.6.2.0 [9] to facilitate coding and categorizing. The various codes were compared based on differences and similarities and sorted into categories. Finally based on content analysis, the underlying meaning that is the latent content of the text was formulated under each of the categories [10]. The results contain direct quotes from participants and narrations are reported as spoken by participants without editing the grammar to avoid losing of the meaning. Quotes that best described the various categories and expressed what was said frequently in several groups were chosen.

Ethical clearance was obtained from Research and Ethics Committee of School of Public Health, College of Health Science of Addis Ababa University. Written permission letter was also produced from Tigray Regional Health Bureau and then from Adigrat Woreda Health Office. Written informed consent was obtained from the study participants. Confidentiality of the participants was kept throughout the study.

## 3. Results

### 3.1. Sociodemographic Characteristics of the Participants

Four FGD sessions were conducted. Thirteen of the participants were in the age group of 25–29 years. Almost all of the participants were Christian Orthodox. More than two thirds of the participants were housewives by occupation. Thirteen of the participants had 1-2 numbers of births. Twenty-three of the participants were modern contraceptive users, which was dominated by Depo-Provera (Table 1).

Six in-depth interviews with FP service providers in public and private health institutions were conducted. All of the participants interviewed were female diploma nurses. Four of the interviewees were trained on insertion and removal of implants and IUD.

### 3.2. Participants Knowledge on LAPMs

Majority of the participants in the FGD were able to identify short-term (Depo-Provera, pills, and condom) and long acting contraceptive methods (implants and IUD). But most of the participants were not able to identify permanent contraceptive methods (male and female sterilization) as contraception. Both women and men in the FGD lack specific information on how LAPMs work especially permanent methods of contraception.

 
*“I did not hear about sterilization of female as well as male before.”  *(30 years old woman, illiterate, para five, Depo-Provera user)
 
*“I heard the presence of medication which is inserted in to hand. But we do not know about male contraceptives. I suggest that it is good if we know and use it.”  *(30 years old man, grade 7 completed)
 
*“I am government employee. There are also other government employees in my neighbor. We did not have knowhow about male contraceptives. Clearly today's discussion is a good opportunity of education for me. I never learnt before as today, because nobody teaches us about such things…” *(37 years old man, Diploma)

The in-depth interviewees indicated that the knowledge of the women on LAPMs is superficial, limited to identifying the methods name. The women lacks deep knowledge of each contraceptive choice especially LAPMs. This is explained by the quality of FP counseling service provided by the providers. They said that the quality of counseling service they provide is not inclusive of all contraceptive choices.

 
*“Not knowing is a problem. I have worked several years even in family planning clinic. Women do not know IUD. They consider as new thing. The knowledge on long acting contraceptives is low. They consider as a new program except pill and injection. The problem is with the health provider. We (health providers) do not explain well to them on each contraceptive method. We only provide what they said; Depo-Provera or pill. We do not properly counsel the mothers for the long acting contraceptives…”  *(IDI 01)

### 3.3. Participants' Perception on LAPMs

Women perceive LAPMs differently. Many of the participants have concerns about implants and IUDs. They had concern about negative effect on the return of fertility after taking implants or IUD. They had fear on the insertion and removal procedures and effect on physical activities. Some of them perceive that these contraceptive methods need eating special food. Some women had expressed their concern on IUD on the need of vaginal examination, discomfort during sex, side effects, and lack of protection against sexual transmitted infections.

 
*“Implant has problem in your daily activity. You can't wash cloths and carry jar (jerican) containing water. You can be harmed if you use it.”  *(24 years old woman, 9th grade completed, Depo-Prevera user)
 
*“Implant has difficulty in doing hard works which results to numbness of your hand. But I did not know why this is happening. Implant has a problem with work especially it is not good for women living in the village working a lot. But this is different in different women.”  *(28 years old woman, 4th grade completed, implanon user)

They told stories about difficulty of removal and resistance to remove implants by the FP providers.

 
*“They (FP providers) did not remove timely at the time you want to remove. They say you will relieve from your pain and stay with it. They did not remove before six months. I know one lady; she is my neighbor, she had visited them repeatedly for removal. She had suffered because they did not obey to remove while she wants to remove it.”  *(36 years old woman, illiterate)

The FP providers stated that there are fears and rumors raised by the women about LAPMs. Women prefer short-term methods. Most of the customers had fear of procedures during insertion and removal and fertility return after the use of implants and IUD. Limiting physical activity after implant insertion is another mostly stated fear by the clients. Women did not trust FP providers on removal of implants.

 
*“Long acting is perceived by the mothers as it needs surgical procedure (operation). They fear it. Implant can cause numbness of the hand, it can pierce you, and you can't work while the implant is in your hand. They perceive IUD as it can go to your head, and not good during sex. Female sterilization they consider as a heavy and difficult procedure and they hate it. It is rarely done except during the labour in the OR. We do not do here. Male sterilization is not talked here. I face only one male who come for sterilization and he returned back because we have no service here. They say it causes impotence. They did not know the access of long acting methods. They know only pill and injection but even though implant last long; they consider as new thing”*. (IDI 01)

### 3.4. Contraceptive Method Preference

The FGD and in-depth interview result revealed that Depo-Provera and pills were considered as normal contraceptive methods. These methods were the most known contraceptive methods. Specifically, injection (Depo-Provera) was perceived as easy to use and stop, easy accessible, and free from any procedures.

 
*“Mothers prefer Depo-Provera and pill because they can stop themselves. They did not need procedure and support from others to stop or remove like implant and IUD.”  *(IDI 02)
 
*“Me myself I have used injection (Depo-Provera) for seven years, I did not face any problem. It is very good.”  *(38 years old woman, illiterate, Depo-provera user)

Most of the participants in the in-depth interview stated that mothers prefer short-term methods (Depo-Provera and pills). The reason they had stated is that the quality of counseling service provided by the FP providers is not inclusive of all contraceptive choices. The fear of procedures for LAPMs is another reason pushing mothers to prefer short-term methods of contraceptives. They do not need any procedures.

 
*“Mothers prefer injection because it is for three months. They (clients) say, I can stop at any time I like. If I face side effect; I can stop it. If I face bleeding; I can stop it. They have fear of resistance by the FP providers to remove the long acting methods. After insertion, they return repeatedly for removal. First the mother should be counseled well, she should also have freedom of decision on removal. They should know what the government bring for them and perceive as their own. But what they usually complain is bleeding, body weakness, hair loss, behavior change because of the implant. Those who took injection when we counsel about long acting contraceptives, they said I will think for that latter but for now I should take Depo-Provera. But usually they did not prefer it latter also.”  *(IDI 03)
 
*“Injection is considered as normal because it is well known whereas long acting is new; but it will be popular as injection in the future. Therefore, if it is introduced well; it will be used by everybody”*. (29 years old woman, 7th grade, jaddle user).

### 3.5. Quality of Family Planning Counseling

Family planning providers in the in-depth interview indicated that the counseling service they were providing was not inclusive of all modern contraceptives. Women, visiting the clinic, had a preference for a type of contraception and they provide what women asked for. They had also indicated the need of training of a team of health providers on LAPMs.

 
*“In our clinic we have provided a lot of long acting contraceptives, almost one per day for long acting contraceptives. I want to say that the health professionals should educate the community with the focus on long acting contraceptives. If you educate appropriately they can easily change and use long acting contraceptives. Because of knowledge women prefer short term but we should not give her simply what she say, we should educate and counsel, then they can change. We have clients as a sample in the Kebeles. They are long acting contraceptives users. They educate women and they bring to our facility for the service.”  *(IDI 041)
 
*“… all professionals should get training on long acting contraceptives. Otherwise there is pressure on one provider to do all the counseling, insertion and removal. This may not be effective. If every health professional have got training on these methods; the workload will be shared and clients will be served well.”  *(IDI 042)
 
*“There is no good knowhow, we are too much busy. We have problem in counseling because of the work load otherwise if we properly counsel the women; they can choose long acting contraceptives. I recommend that the community leaders and religion leaders can support in teaching the whole community.”  *(IDI 05)

## 4. Discussion

This study revealed that the knowledge on LAPMs is limited to recognizing the name. Most participants are not able to identify permanent contraceptive methods. They lack knowledge on how these methods work and how they can use them especially concerning permanent methods of contraception. Most of the participants have misunderstanding and fear of procedures. They had expressed their concern on the return of fertility after using implants or IUD. They had fear of resistance of the providers on removal of implants up on their demand to remove. These perceptions can be a barrier to use these methods. The FP providers indicated the presence of fears and rumors, raised by their clients about each method, and mistrust towards the providers on removal especially implants. The fear came from their own and their peers' experiences. Mothers prefer to use methods without any procedure. The providers stated their weakness on counseling of all contraceptive choices. The FP counseling service is not comprehensive of all modern contraceptive methods, especially LAPMs. Short-term contraceptives (Depo-Provera and pills) were the most commonly known and preferred methods. Injection (Depo-Provera) was perceived as a common and normal method. This avoids the fear of resistance to remove implants and IUD by the FP providers. The providers stated that demand for LAPMs is increasing. But the inclusion of LAPMs especially permanent methods (tubal ligation, and vasectomy) in the counseling of methods was still not well done.

In interpreting these results, there are certain limitations with the study design that might impact upon the conclusions drawn. First and foremost, the purposive sampling may be biased by including individuals who are more comfortable talking about family planning matters. Besides other acknowledgeable limitations associated with the use of qualitative methodology, the result may not be automatically transferred to other countries with different cultural contexts. The strength of this study lies in its richness of data, including women, men, and FP providers, which ensured enough data triangulation and saturation.

Knowledge on different contraceptive methods is the key to choose the contraceptive method and to practice it [11, 12]. Almost universal awareness of at least one modern contraceptive method was observed in the studies conducted in Southern Ethiopia [13, 14]. But this finding may have been the result of knowledge of a method variable that was evaluated as “have you heard about it”, which may not actually reflect to an adequate knowledge to the method in question. Permanent contraceptive methods were the least known method in the Ethiopian Demographic Health Survey 2011 and study conducted in Mekelle [4, 5].

Some women perceived that contraceptives interfered with fertility, and they were scared to use something that could harm their ability to reproduce [7, 15–18]. The experiences of other people on specific contraceptive method strongly affect the choice and perception of other women [19]. More than one quarter (29.7%) of married women perceive that IUD insertion procedure can result in shame [5]. Some women perceive IUD as it can limits day to day activities, and female sterilization is dangerous [5]. Negative provider attitudes, misinformation on the part of providers and high service charges were identified as barriers to FP use in Pakistan [8].

This could be some of the factors responsible for low utilization of LAPMs. Every woman who seeks FP information or services should be educated and counseled on all choices of modern contraceptive methods; and given an opportunity to ask questions after the provider has described the methods available. This can help to avoid the fears and misperceptions hindering LAPMs use. Educational and enlightenment programs should focus on providing specific knowledge, with special attention to correcting common misconceptions about the methods. Health care providers should be encouraged to inform all potential users about the methods, and to prescribe it to clients who require it [20].

## 5. Conclusion

This study is carried out to explore the knowledge and perceptions of married women and men on LAPMs. Knowledge of specific methods is very low particularly of long-term and permanent methods of contraception. Therefore, there is need to broaden the knowledge of such methods of family planning. There are personal barriers and knowledge gaps on LAPMs. Most of the participants had misconceptions and fears on LAPMs. Family planning counseling service was not inclusive of LAPMs. Improving the counseling service program can help women to increase the knowledge of each contraceptive choice and choose appropriate contraceptive methods and use them consistently. Women need more information from their health care providers in order to make informed choices about their FP preferences. Therefore, the FP providers should counsel on all of the FP methods, address misconceptions and fears that exist about LAPMs, and highlight the benefits of LAPMs during FP counseling. The FP counseling should also include the benefits, mechanism of action of modern contraceptive methods, and side effects and how to manage them. Teams of health care workers should be trained to offer the service, not just individuals within health centers and clinics. There should be strategies to increase male participation in family planning activities. Further detailed investigation of the quality of counseling service in the town should be conducted.

## Figures and Tables

**Table 1:** Sociodemographic characteristics of FGD participants, Adigrat town, January 2012 (*n* = 32).

Sociodemographic characteristics	Frequency (*n*)
Sex	
Female	25
Male	7
Age (years)	
18–24	6
25–29	13
30–34	4
35+	9
Religion	
Orthodox	30
Catholic	1
Muslim	1
Educational level of participant	
No education	9
Primary (1–8th)	12
Secondary (9–12th)	7
Higher education	4
Occupation of participants	
Housewife	22
Employed (government and private)	6
Daily labourer	4
Number of births	
0	3
1-2	13
3-4	7
5+	9
Contraceptive use status	
Nonuser	9
Pill	6
Depo-Provera	12
Implants	5
